# Supplementary material for: From months to minutes: Creating Hyperion, a novel data management system expediting data insights for oncology research and patient care
Source: PLOS Digit Health. 2022 Nov 1;1(11):e0000036. doi: 10.1371/journal.pdig.0000036 (PMC9931228; doi:10.1371/journal.pdig.0000036)
Supplement: S1 Text — Fig A. Nursing dashboard. Fig B. Example of Provider Dashboard landing page, with interactive features (hover-over pop-up text and ability to click on bar graphs to “drill down” on data). Fig C. Clinical Trial dashboard main page, with interactive features. (DOCX) [file pdig.0000036.s001.docx]

**SUPPORTING INFORMATION: S1 Text**

*Logo removed at request of journal*


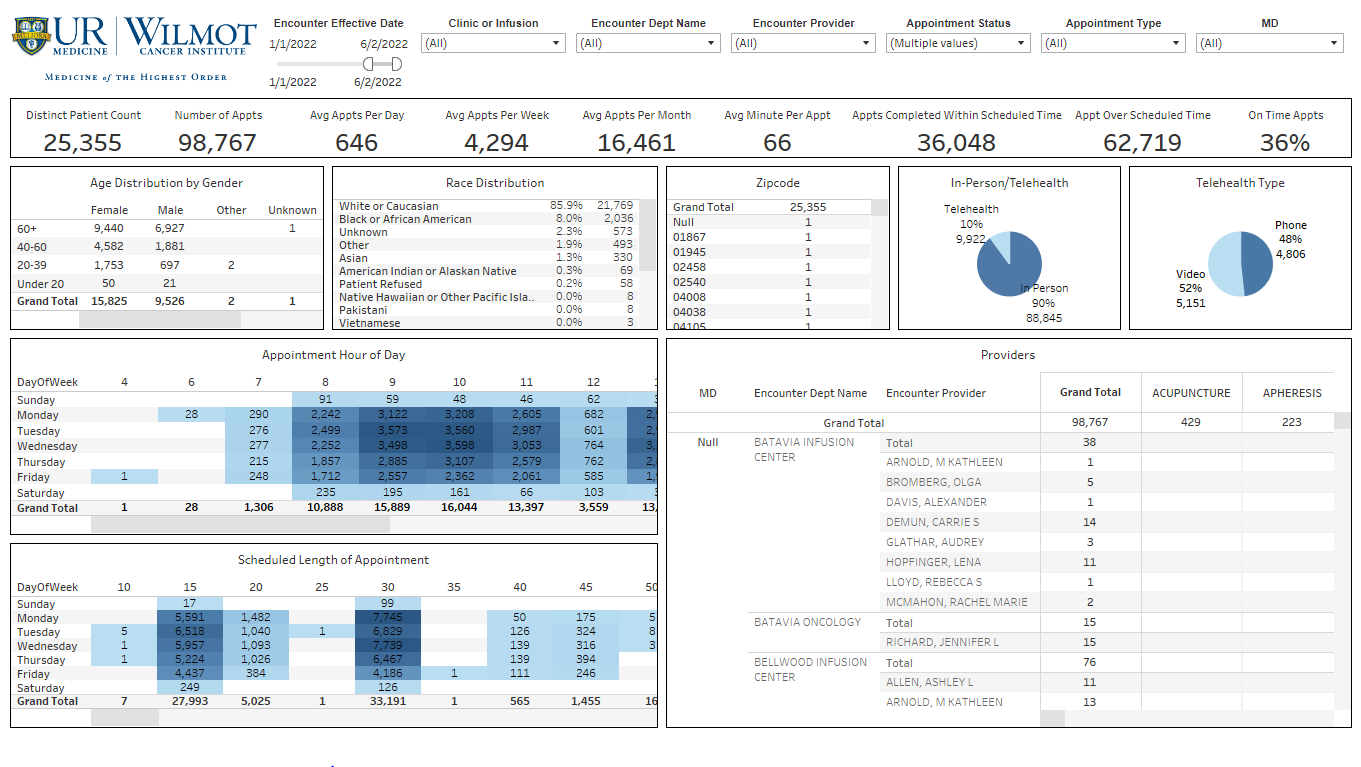
**Fig A.** Nursing dashboard.


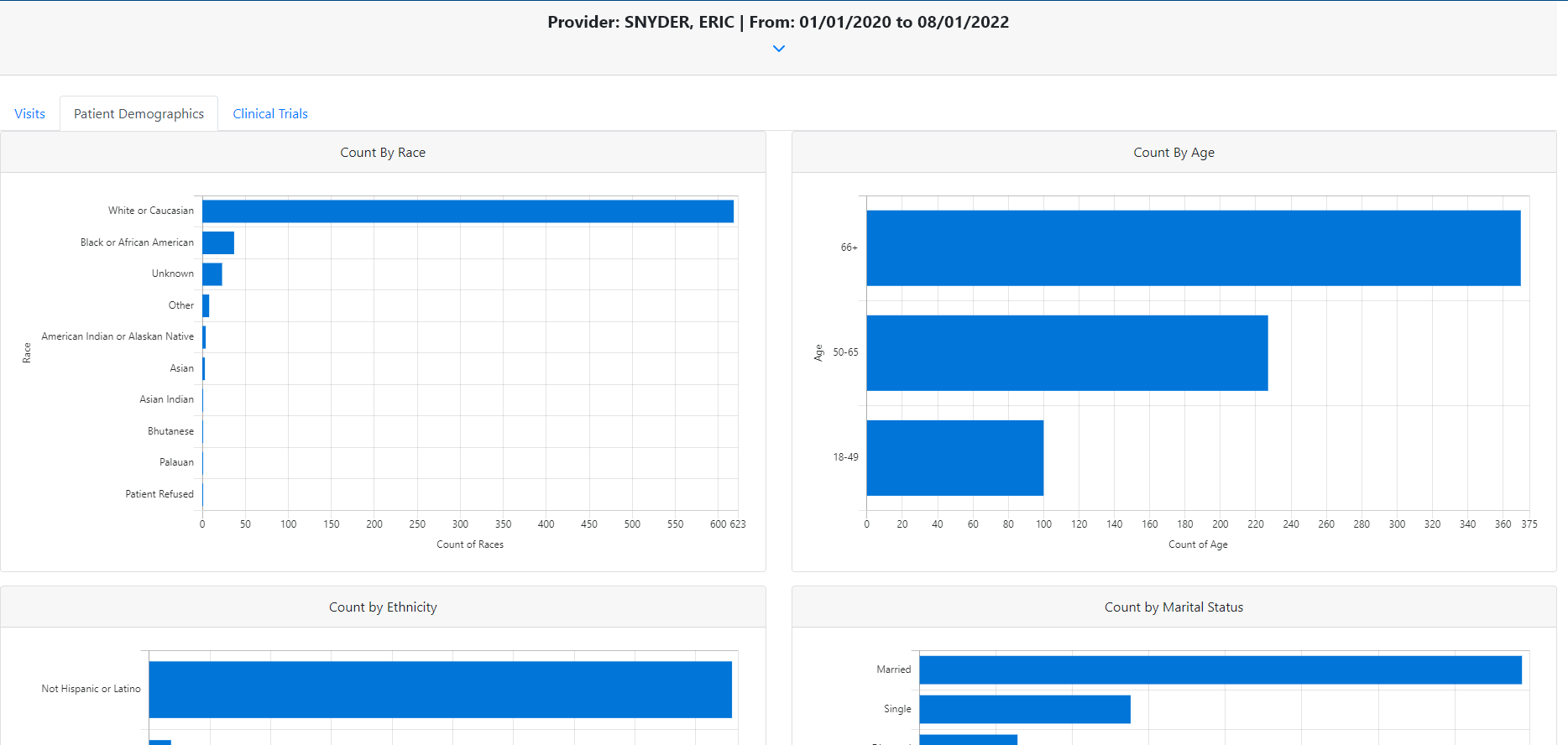


**Fig B.** Example of Provider Dashboard landing page, with interactive features (hover-over pop-up text and ability to click on bar graphs to “drill down” on data).


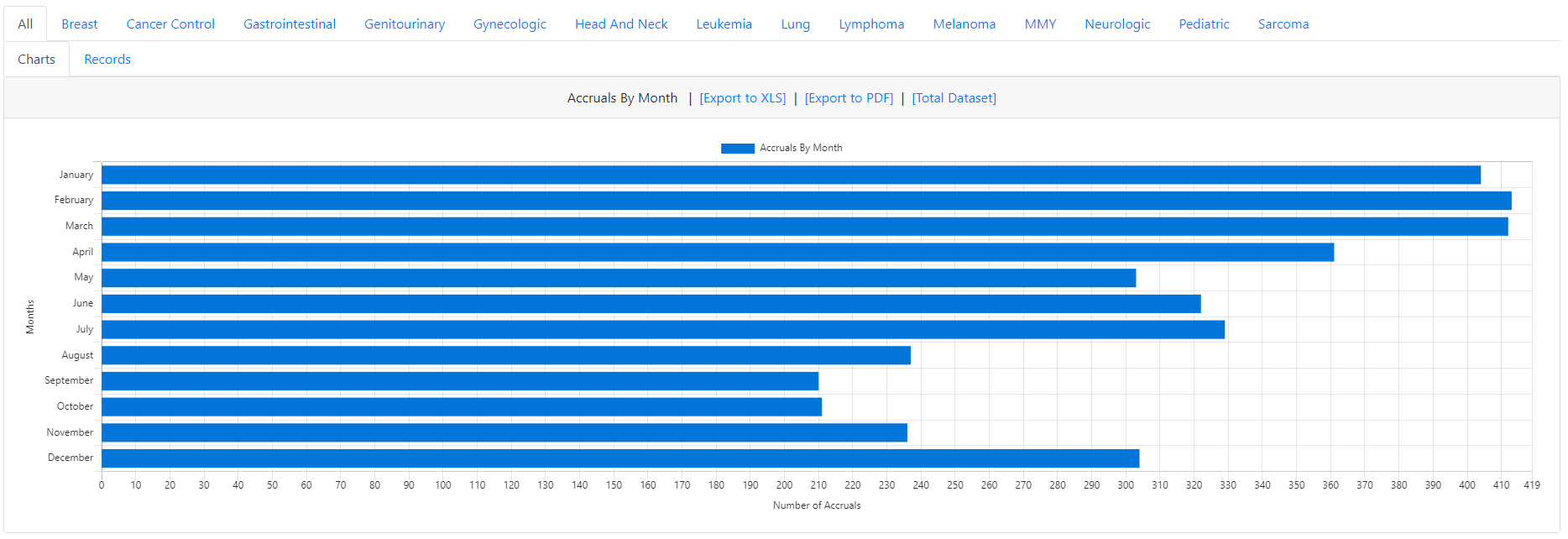


**Fig C.** Clinical Trial dashboard main page, with interactive features.
