# Supplementary material for: From months to minutes: Creating Hyperion, a novel data management system expediting data insights for oncology research and patient care
Source: PLOS Digit Health. 2022 Nov 1;1(11):e0000036. doi: 10.1371/journal.pdig.0000036 (PMC9931228; doi:10.1371/journal.pdig.0000036)
Supplement: Attachment — Submitted filename: PLOSdigital_Hyperion_responsetoreviewercomments.docx [file pdig.0000036.s002.docx]

August 18, 2022

Re: Manuscript revision for PLOS Digital Health

Dear Reviewers:

Thank you for your time and effort in reviewing our manuscript entitled, “From months to minutes: creating Hyperion, a novel data management system expediting data insights for oncology research and patient care.” We appreciate the opportunity to submit a revised manuscript. The reviewer comments are duplicated below, with our responses to each. A manuscript with “track changes” enabled has also been submitted to highlight the changes.

***Reviewer 1***

*This paper highlights the design and implementation of an in-house customized data management software in an academic medical center involving a small, cross-functional team. This is an excellent practical project and seems to work well for a specific context. However, for digital health and health informatics, the methods and measures are unclear and don’t have adequate rigor. I am also unsure about the generalizability of the study findings.*

Thank you for this insightful comment. We concur that this manuscript describes a pragmatic and contextual software implementation rather than a generalizable implementation study. With that in mind, our focus was not on specific and rigorous methods and measures but on a narrative description to provide multi-level process insights. We address this comment with more specificity below.

*1. Some of the challenges that have been identified are very specific to a context. It would be helpful to address the generalizability of these challenges to diverse cancer settings (academic and community centers). Also, what methods were used to determine these challenges? How did the authors ascertain that these were the “only” challenges?*

Thank you for this comment. We did not assess how or to what extent challenges are shared across other sites for the specific purposes of this manuscript, nor were these generated through qualitative methodologies most used in healthcare (e.g. focus groups, Delphi). These challenges emerged from the iterative design process itself as well as our team’s combined experience working in multiple cancer centers, and we included them to provide an insight into this process and to provide narrative structure to the manuscript. They are therefore not the “only” challenges, but should be understood more as a framing device. We added this sentence: “These challenges, abstracted from our iterative design process including input from all stakeholders, are distilled principles which could be considered across diverse implementations, rather than a comprehensive description of the discrete implementation challenges we faced.”

*2. Also, how were the design elements for each challenge developed. Replicability of these findings is difficult, and this study seems more like a solution to a contextual problem.*

We do not consider this to be a study, but rather a high-level description of a pragmatic implementation. Given the broad level of abstraction of the identified challenges/principles, we consider it highly likely that they are preserved across a variety of sites. Table 1 summarizes the design elements corresponding to each challenge – again, to give narrative structure and coherence to our overall process – but describing the development of each design element is beyond the scope and intent of this paper. We acknowledge that specific design decisions and elements, as with other complex data architecture implementations, are unlikely to be strictly reproducible across sites.

*3. It is also clear if any user feedback on ease of use was measured. Also, were the end-users involved in design and development of Hyperion? Please include more details. The authors have included usage metrics and these metrics would often cloud the overall usability and end-user preferences.*

Thank you for this comment. Our methods are adapted from industry software design and development processes. Feedback was collected from users throughout the development process, albeit not within a structure typically utilized in healthcare implementations.

We highlighted the role of our project manager and added the following sentence to methods: “The design and development process was conducted using Project Management Body of Knowledge (PMBOK) best practices under the guidance of the project manager, including scoping, planning, identifying and meeting with stakeholders for each design phase, and communicating requested changes to the development team.”

*4. The authors make a case for a successful implementation of custom data management software but haven’t measured any implementation outcomes.*

Thank you for this comment. We quantify total cost, processing capacity, user base, and response times, which we hope allows for some conclusions to be drawn.

This is a pragmatic implementation adapting industry best practice to an academic setting, and not a research study, so we felt that adopting language of a research paradigm (e.g., outcomes) was not appropriate for our manuscript. However, we do think that measures often included as “implementation outcomes” can be deduced from the paper: for example, adoption, appropriateness, costs, feasibility, and sustainability are all measured, but we do not consider it within the scope of this paper to describe them as outcomes within an implementation science paradigm, since this was not the governing paradigm of our process.

*5. It is not clear what were the novel design ideas in data architecture.*

Thank you for this comment. In general, the architecture elements themselves are not novel, which we believe is important to highlight for reproducibility and scalability assessments. The breadth of capabilities is novel, replacing what would normally require multiple software systems. Hyperion contains a federated data warehouse as well as dashboarding, data analytics capacity, enhanced security, sandboxing, and integrated ticketing and governance. The attention to “lowering the skill floor” throughout the software elements is also novel, allowing updating and maintenance by nonexperts. Creating new interfaces, for example, is achievable without programmers in most cases and for most data models and formats. The use of multi-threaded processing implemented throughout Hyperion allows for future addition of analytic pipelines, including for genomic and other “big data” sources. These elements are presented within the results, and we have added a table (Table 4) comparing Hyperion to other commonly available clinical data management software.

We added the following sentence to results: “This design also facilitates future implementation of analytic pipelines, including machine learning pipelines.”

*6. It would be helpful to also provide a rationale on why an in-house customized data management software was required and provide some generalizable knowledge on how academic medical centers should take decisions for or against the development of in-house customized data management software. Much of the limitation section highlights all the issues on why academic medical centers are unwilling to support in-house software development for patient data and it could be that this is a “unique” situation. The generalizability of this study findings is what I am struggling most. This is an excellent practical project with great value to a specific context. However, it lacks rigor in methods, measures, and generalizability.*

Thank you for this excellent comment, and we agree that the rationale is both interesting to describe and likely highly variable across sites considering this approach. We would consider advice on how to make this decision beyond the scope of this paper, as our intention is to describe a single-site implementation with intention to multiple cognitive process and design levels (i.e., design elements, organizational structures, and guiding principles). Our intention is descriptive rather than prescriptive. As this is not a research study, we did not adopt language from that paradigm (rigor, measures, generalizability).

*1. Figure 5 is titled most accessed pages by end users through October 2021. Instead of merely presenting the pages from 1-6 ( clinical trial dashboard- Referrals), it would be helpful if the number of times each of these pages have been accessed per a specific time period or the average number for a defined time period.*

This is an excellent suggestion and create a more informative visual. We removed Figure 5 and created a table showing the suggested data (Table 3).

***Reviewer 2***

*References could be added to several statements in the manuscript. Regarding related work, the authors only mention one institution (reference 4). This could be extended to include more examples since the authors refer to “Some institutions”.*

Thank you for this comment. As research data warehouses are now quite common, we decided to eliminate the reference to a single implementation. Instead, we added two references (Lyman et al and Sheta et al) describing data warehouse implementations, including in a cancer center. We also highlighted how our implementation differs from other commonly available clinical data management approaches.

*It would be interesting to visualize graphical examples of tasks or dashboards which are possible with Hyperion as described on the paper, as supplementary material.*

Thank you. We will add example dashboard views to the supplementary material; however, as many of the dashboards are interactive, it is difficult to convey the impact of these dashboards. We are happy to offer demonstrations to readers on request, if this can be added somewhere within the manuscript materials.

*Please check the references in the manuscript and include those currently after the dot before the final dot, examples in “while adhering to already thin financial budgets.[2]” and “skilled personnel.[5]”.*

Thank you for this comment. We have corrected citation formatting.

*The first sentence in Data Governance section should be divided into at least 2 sentences.*

We modified the sentence to enhance clarity:

“Data governance processes follow a centralized data governance model with a decentralized execution standard: this allows centralized control and authority to reside with the informatics team but permits the individual users and groups to be able to execute queries and analytics on curated sandboxed data sets or dashboards.”

*In Usage metrics specify the start and end dates, instead of ‘through October 2021’, so that the reader understands the referred time period. Figure 5 legend should also include this information.*

Thank you. We have updated the usage metrics, and replaced Figure 5 with Table 3 as described above.

*In Figure 1 please add the acronym meaning for your center WCI.*

Addressed in legend.

*Figure 2 is missing y and x axis legends.*

Thank you. These have been added.

***Reviewer 3***

*Please follow the submission guidelines of the journal (https://journals.plos.org/digitalhealth/s/submission-guidelines):*

*- Abstract is conceptually divided into three sections (Background, Methodology/Principal Findings, and Conclusions/Significance), do not apply these distinct headings to the Abstract within the article file.*

Abstract headings removed and abstract slightly modified; word count is within requirements.

*- Include an 150-200 word author summary*

This has been added below the abstract.

*- Word count: according to the submission guidelines, there is no limit of 4000 as mentioned in your paper*

Word count updated.

*The background information is quite limited. What are state-of-the-art Clinical Data Management (CDM) systems? How does Hyperion compare to these? It might be good to include a table with features, comparing current CDM systems.*

This is an excellent suggestion. Many of the available CDM’s are commercial software rather than open source implementations; it is outside the scope of this paper to review their features, and this may be inappropriate for an academic journal. However, we did create a table (Table 1) comparing Hyperion features to those of commercially available CDM software.

*The number of references (7) is very small. Sites/software such as REDCap, OnCore, clinicaltrials.gov should be referenced, using a scientific publication (e.g. REDCap:* [*https://www.sciencedirect.com/science/article/pii/S1532046408001226*](https://www.sciencedirect.com/science/article/pii/S1532046408001226)*).*

Thank you for this comment. References have been added. We removed the name of the proprietary clinical trial software (OnCore).

*It's surprising that a paper about a clinical data management platform does not discuss the FAIR Guiding Principles (https://www.nature.com/articles/sdata201618). How does Hyperion handle these four aspects?*

Thank you for this comment. We added an explicit reference to FAIR principles under Discussion: “Furthermore, Hyperion aligns with FAIR data principles (findability, accessibility, interoperability, and reusability).”

Although the alignment with these principles is implicitly referenced throughout the manuscript, we attempted to make these connections more explicit. We added the following within Results:

1.	Interoperability: “…bidirectional interfaces can be easily configured for all common data formats and models (HL7 FHIR, XML, API etc.).”

2.	Reusibility: “As all data tables are normalized and explicitly defined within our system, data reusability across the system is also facilitated.”

We hope that the connections with the first two principles are already recognizable within the manuscripts.

*The Hyperion front-end works with HTML5 and JavaScript. Was it tested in all major browsers (Chrome/Firefox/Edge/Safari/Opera)?*

Yes. We added the following sentence to results: “The system has been tested and functions with all major web browsers.”

*Probably the most difficult task in a custom-made CDM system is security. I miss the details on security in this paper. For example, does Hyperion support two-factor authentication?*

This is an excellent comment; thank you. Yes, Hyperion supports multi-factor authentication. We added a section to results titled “Security”: Hyperion is only accessible via a secure networked computer or institutional virtual private network (VPN). Hyperion’s custom security module augments the institution’s Active Directory (AD) authentication and allows for user auditing and access control down to the individual application level. This permits user access to be customized by role and job function. and allows for the capture of full details on each user action, which Hyperion logs and monitors. Secure sandboxes are provided to users for data analytics purposes, without the ability to download data; this method of viewing and analyzing Hyperion reports is strongly encouraged. Otherwise, requested data documents are sent via either end-to-end encrypted email or secure file transfer protocol (SFTP). All documents able to be downloaded by the user include meta tags to identify the source of download, the individual user, and date/time stamps. In the event of a data consistency issue, the document can be compared to the logged SQL audit calls stored within Hyperion to ensure data was not altered. Request fulfillment for patient-identifiable data meets all institutional data security policies, including review by the Data Governance Committee.

*Is there a way to access imaging data (e.g. DICOM) through Hyperion, for example in the Physician Dashbord? The same for genomics data, digital pathology data, etc.: does Hyperion contain a link to these raw data?*

Thank you. Yes, Hyperion contains DICOM imaging links, though we do not store actual imaging files on the Hyperion server. Similarly, API’s to genomic and molecular datasets (e.g., FoundationOne data and researcher datasets) are integrated, though these are not yet directly accessible via dashboards. The data are available by request as described in the Data Governance and Security sections, under Results.

We amended a sentence in Results: “Hyperion’s analytics-processing framework enables real-time analytics across any data element in the system, including those accessed via Application Programming Interfaces (APIs, e.g., imaging, pathology, and molecular/genomic data).”

*A screenshot of one of the other dashboards than just CANVAS would be useful, for example the Clinical Trials Dashbord or Physician Dashboard (dummy data could be used), since these are most accessed?*

We have included basic screenshots in the supplementary material; as stated above, the visualizations do not convey the interactivity and ability to drill down on these data.

Again, we appreciate your time, effort, and insight; your comments have significantly improved our manuscript.

Sincerely,

Erika Ramsdale, MD

Corresponding author
